# Supplementary material for: The Association between Prognostic Nutritional Index (PNI) and Intraoperative Transfusion in Patients Undergoing Hepatectomy for Hepatocellular Carcinoma: A Retrospective Cohort Study
Source: Cancers (Basel). 2021 May 21;13(11):2508. doi: 10.3390/cancers13112508 (PMC8196581; doi:10.3390/cancers13112508)
Supplement: Supplementary file 1 [file cancers-13-02508-s001.zip › cancers-1198428-supplementary.pdf]

Table S1. Univariate and multivariable analysis of the risk Factors of postoperative transfusion.

	Univariate			Multivariate		
	**OR**	**95% CI**	**P–value**	**OR**	**95% CI**	**P–value**
PNI (< 44)	2.41	1.20–4.83	0.014	1.92	0.90–4.10	0.089
Age	0.99	0.96–1.03	0.727			
Sex (male)	1.32	0.59–2.98	0.499			
BMI	0.93	0.82–1.05	0.242			
DM	0.45	0.06–3.40	0.445			
HTN	1.35	0.40–4.54	0.624			
MELD scores	1.37	1.09–1.71	0.007	1.27	0.98–1.64	0.066
CTP scores	2.15	1.23–3.75	0.007			
TNM staging			0.070			
1	1.00(Ref.)					
2	3.73	1.32–10.57	0.013			
3	4.40	0.94–20.51	0.059			
4	1.45	0.65–3.24	0.362			
Hemoglobin	0.84	0.68–1.03	0.100			
RDW	1.20	1.02–1.42	0.030			
Operation time; min	1.01	1.01–1.01	< 0.001	1.01	1.01–1.01	< 0.001
Synthetic colloid use	2.38	1.12–5.05	0.024	1.97	0.90–4.30	0.090
Extensive surgery (≥3 segments)	1.60	0.37–6.92	0.529			
Laparoscopic surgery	0.63	0.24–1.65	0.347			

OR, odds ratio; CI, confidence interval; BMI, body mass index; DM, diabetes mellitus; HTN, hypertension; MELD, model for end-stage liver disease; CTP, child-turcotte-pugh; RDW, red cell distribution width; PNI, prognostic nutritional index; SD, standard deviation. Values are expressed as the mean ± SD, median (interquartile range), or n (proportion).
